# The Impact of Timing of Stent Removal on the Incidence of UTI, Recurrence, Symptomatology, Resistance, and Hospitalization in Renal Transplant Recipients

**DOI:** 10.1155/2021/3428260

**Published:** 2021-07-02

**Authors:** Ziad Arabi, Khalefa Al Thiab, Abdulrahman Altheaby, Mohammed Tawhari, Ghaleb Aboalsamh, Mohamad Almarastani, Samy Kashkoush, Mohammed F Shaheen, Abdulrahman Altamimi, Lina Alnajjar, Rawan Alhussein, Raghad Almuhiteb, Bashayr Alqahtani, Rayana Alotaibi, Marah Alqahtani, Yahya Ghazwani, Wael O'Hali, Khalid Bin Saad

**Affiliations:** ^1^Division of Adult Transplant Nephrology, King Abdulaziz Medical City, Riyadh, Saudi Arabia; ^2^King Abdullah International Medical Research Center, Riyadh, Saudi Arabia; ^3^College of Medicine, King Saud Bin Abdulaziz University for Health Sciences, Riyadh, Saudi Arabia; ^4^Pharmaceutical Care Department, King Abdulaziz Medical City, Riyadh, Saudi Arabia; ^5^Department of Hepatobiliary Sciences and the Organ Transplant Center, King Abdulaziz Medical City, Riyadh, Saudi Arabia; ^6^Department of Pharmacy Practice, College of Pharmacy, Princess Nourah Bint Abdulrahman University, Riyadh, Saudi Arabia; ^7^College of Pharmacy, Princess Nourah Bint Abdulrahman University, Riyadh, Saudi Arabia; ^8^Division of Urology, King Abdulaziz Medical City, Riyadh, Saudi Arabia

## Abstract

**Purpose:**

To evaluate the impact of early (<3 weeks) versus late (>3 weeks) urinary stent removal on urinary tract infections (UTIs) post renal transplantation.

**Methods:**

A retrospective study was performed including all adult renal transplants who were transplanted between January 2017 and May 2020 with a minimum of 6-month follow-up at King Abdulaziz Medical City, Riyadh, Saudi Arabia.

**Results:**

A total of 279 kidney recipients included in the study were stratified into 114 in the early stent removal group (ESR) and 165 in the late stent removal group (LSR). Mean age was 43.4 ± 15.8; women: *n*: 114, 40.90%; and deceased donor transplant: *n*: 55, 19.70%. Mean stent removal time was 35.3 ± 28.0 days posttransplant (14.1 ± 4.6 days in the ESR versus 49.9 ± 28.1 days in LSR, *p* < 0.001). Seventy-four UTIs were diagnosed while the stents were in vivo or up to two weeks after the stent removal “UTIs related to the stent” (*n* = 20, 17.5% in ESR versus *n* = 54, 32.7% in LSR; *p*=0.006). By six months after transplantation, there were 97 UTIs (*n* = 36, 31.6% UTIs in ESR versus *n* = 61, 37% in LSR; *p*=0.373). Compared with UTIs diagnosed after stent removal, UTIs diagnosed while the stent was still in vivo tended to be complicated (17.9% versus 4.9%, *p*: 0.019), recurrent (66.1% versus 46.3%; *p*: 0.063), associated with bacteremia (10.7% versus 0%; *p*: 0.019), and requiring hospitalization (61% versus 24%, *p*: 0.024). Early stent removal decreased the need for expedited stent removal due to UTI reasons (rate of UTIs before stent removal) (*n* = 11, 9% in the early group versus *n* = 45, 27% in the late group; *p*=0.001). The effect on the rate of multidrug-resistant organisms (MDRO) was less clear (33% versus 47%, *p*: 0.205). Early stent removal was associated with a statistically significant reduction in the incidence of UTIs related to the stent (HR = 0.505, 95% CI: 0.302-0.844, *p*=0.009) without increasing the incidence of urological complications. Removing the stent before 21 days posttransplantation decreased UTIs related to stent (aOR: 0.403, CI: 0.218-0.744). Removing the stent before 14 days may even further decrease the risk of UTIs (aOR: 0.311, CI: 0.035- 2.726).

**Conclusion:**

Early ureteric stent removal defined as less than 21 days post renal transplantation reduced the incidence of UTIs related to stent without increasing the incidence of urological complications. UTIs occurring while the ureteric stent still in vivo were notably associated with bacteremia and hospitalization. A randomized trial will be required to further determine the best timing for stent removal.

## 1. Background

Ureteric stents are shown to decrease post renal transplant urological complications but are believed to increase the risk of urinary tract infections (UTIs) [1–7]. Hence, our practice has changed over the years. Initially, we adopted a protocol where the ureteric stents remained in vivo for 2 months after living donor kidney transplantation (LKT) and 2–3 months after deceased donor kidney transplant (DKT). In 2018, our center modified its protocol of ureteric stent removal to the following:

1. Ureteric stents to be removed “routinely”: within 2–4 weeks posttransplant.
2. Ureteric stents to be removed “expeditiously” if a patient develops UTIs. It is recommended to remove the stent once the infection is controlled.
3. Urinary stent to be removed “emergently” in the case of migrated stent to the urethra or in the case of unstable patients with severe sepsis due to UTIs or in the case of fungal infection [8].

In this study, we aim to examine the impact of protocol change leading to earlier ureteric stent removal on the incidence and characteristics of UTIs post kidney transplantation. Three weeks' timing has been shown in previous studies to reduce the risk of UTIs without an increase in major urological complications and hence was adopted in this study as a cutoff value [5, 9].

## 2. Methodology

After obtaining the institutional board review approval (RC20/138/R), a retrospective study was conducted including adult renal transplant recipients at King Abdulaziz Medical City, Riyadh, Saudi Arabia, from January 2017 to May 2020 with 6 months follow-up. Renal transplant recipients were excluded if they were diagnosed with UTI within one month prior to transplantation or if they experienced early graft failure. All patients received either anti-thymocyte globulin (ATG) or basiliximab plus the standard triple immunosuppressant combination: tacrolimus, prednisone, and mycophenolic acid. Data about patients' characteristics and UTI outcomes were collected, including incidence, risk factors, symptomatology, prevalence of multidrug-resistant organisms (MDRO), rate of hospitalization, and treatment. Owing to the lack of consensus on the best timing for stent removal, we used 3 weeks' time to separate early from late stent removal. The early stent removal groups had the stent removed within 3 weeks from the renal transplant date, while the late stent removal group had the stent removed anytime beyond 3 weeks. We considered UTIs as “stent-related UTIs” if UTIs occurred while the stent is still in place up until two weeks after removing the stent. UTIs were considered caused by MDRO if the causative organism was resistant to at least one agent of three or more antimicrobial categories.

UTIs were classified according to their symptoms into [10–12]:

1. Asymptomatic bacteriuria; >10^5^ colony-forming units (cfu)/mL
2. Simple (uncomplicated) UTI: positive urine culture in addition to any urinary symptoms such as dysuria, urgency, frequency, or suprapubic pain, but no systemic symptoms.
3. Complicated UTI: positive urine culture in addition to systemic symptoms such as fever, chills, flank, and/or allograft pain.
4. Complicated UTI with bacteremia.
5. Recurrent UTI: more than one UTIs in the first 6 months with the same or different microorganisms.

We reviewed the timing of UTIs and compared UTIs before and after stent removal regarding their incidence, recurrence, symptomatology, resistance, type of treatment, and need for hospitalization. We also studied the urological complications in the early versus the late stent removal groups.

### 2.1. Statistical Analysis

All analyses were performed using IBM SPSS software 23.0 (IBM Co., Armonk, NY, USA). Continuous variables were presented as mean (SD). Categorical variables were expressed as proportions (percentages). We compared data using *t*-test, Mann–Whitney *U* test, chi-squared test, or Fisher's exact test as appropriate. We used logistic regression analysis to calculate UTI odds ratio, with adjustments for age and gender. Survival analysis was performed using the Cox regression model and adjusted for age and gender. All statistical tests were two-sided, and *P* values <0.05 were considered statistically significant.

## 3. Results

**Figure 1:**
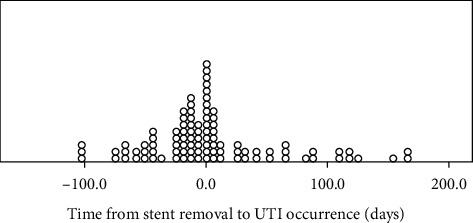
Time from stent removal to UTI occurrence. Most of the UTIs during the first 6 months post renal transplantation are stent related (occurs while the stent in place and up to two weeks post stent removal). After the stent removal, UTIs clearly become sporadic and less frequent.

**Figure 2:**
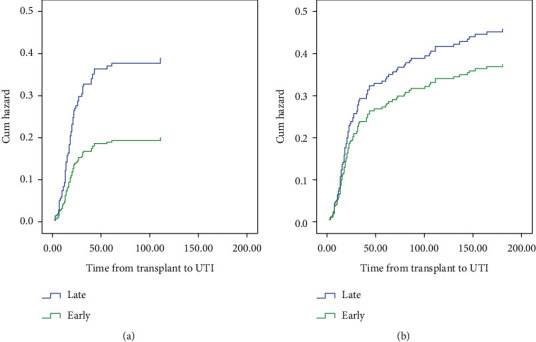
Adjusted Cox proportional hazard ratio. (a) UTIs related to stent. ESR has significantly reduced the incidence of UTIs related to stent (HR = 0.505, 95% CI (0.302 to 0.844), *p*=0.009). (b) UTIs by 6 months. The positive effect of ESR became numerically but not statically significant when reviewing the total UTIs by 6 months (HR: 0.787, CI: 0.474-1.305).

**Figure 3:**
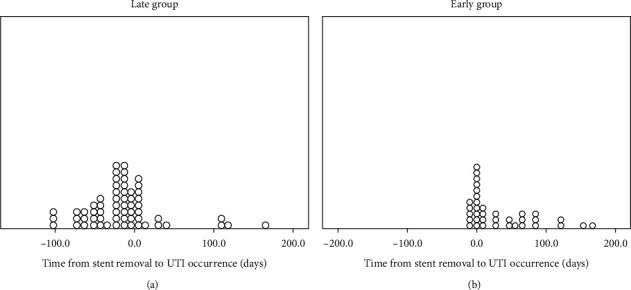
UTI distribution before and after stent removal. (a) In the late stent removal group. (b) In the early stent removal group. UTIs before stent removal were much higher in the late group (*n* = 45, 27% versus n = 11, 9% in the early group; *p* < 0.001).

1. Patients' characteristics: a total of 279 kidney recipients were included in the study (114 in the early group and 165 in the late group). Mean age was 43.4 ± 15.8; of them 114 (40.90%) were women and 55 (19.70%) were deceased donor transplant recipients. Mean stent removal time was 35.3 ± 28.0 days posttransplant (14.1 ± 4.6 days in the early group versus 49.9 ± 28.1 days in the late group, *p* < 0.001) (Table 1).
2. Secondary analysis: the two groups were statistically different with regard to the donor type, serum creatinine at 6 months, and the type of antibiotics prophylaxis used. Secondary analysis showed that these factors were not contributing to the risk of UTIs and recurrence in our study. On the other hand, age >40, female gender, transplantation abroad, and neurogenic bladder were contributing factors of UTIs [(OR: 2.176, CI: 1.187–3.986), (OR: 5.008, CI: 2.74-9.156), (OR: 5.008, CI: 2.607-27.05), and (OR: 5.646, CI: 1.016-31.379), respectively]. These factors were distributed symmetrically between the early and the late groups.
3. Timing of UTIs in relation to stent removal: as shown in Figure 1, most of the UTIs diagnosed during the first 6 months posttransplantation occurred while the stent was still in place extending to up to two weeks post stent removal. Afterward, UTIs become sporadic and less frequent.
4. UTIs and other outcomes in relation to stent removal: there were 74 UTIs occurred while the stent was still in vivo—related to the stent—(*n* = 20, 17.5% in the early group versus *n* = 54, 32.7% in the late group; *p*=0.006). By six months after transplantation, there were 97 additional UTIs (*n* = 36, 31.6% UTIs in the early group versus *n* = 61, 37% in the late group; *p*=0.373) (Table 2).
5. The risk of UTIs in relation to stent removal: ESR significantly decreased the incidence of UTI related to stent (HR = 0.505, 95% CI: 0.302 - 0.844, *p*=0.009). The positive effect of ESR became numerically but not statically significant when reviewing the total UTIs by 6 months (HR: 0.787, 95% CI: 0.474-1.305).  Figure 2 shows the adjusted Cox proportional hazard ratio of UTIs in both groups.
6. Further comparison was done in regard to the incidence, distribution, and symptomatology of UTIs before and after stent removal in the (late versus early) stent removal groups. And the results are shown in Figure 3 and Table 3.
7. UTIs before stent removal were much higher in the late group (*n* = 45, 27% in the late group versus *n* = 11, 9% in the early group, *p* < 0.001) (Figure 3). UTIs before stent removal are considered as an indication for “expedited” stent removal as per our center protocol (Figure 3).
8. In addition, UTIs before stent removal when compared to UTIs after stent removal were more complicated (17.9% versus 4.9, *p*: 0.019) and more associated with bacteremia (10.7% versus 0%; *p*: 0.019) and more associated with hospitalization (61% versus 24%, *p*: 0.024). UTIs before stent removal also recurred at higher numerical rate (66.1% versus 46.3%; *p*: 0.063) (Table 3).
9. Of note, only one of the 6 patients who underwent emergent stent removal for non-UTIs reason developed UTIs (5 cases versus one, *p*: 0.048) as shown in Table 1. The incidence of MDRO in the early versus late stent removal groups was (33% versus 47%, *p*: 0.205), as shown in Table 3.
10. When comparing the odds ratio of UTIs related to stent in the relation to the timing of stent removal, it is noted that removing the stent before 21 days posttransplantation decreased UTIs related to stent (aOR: 0.403, CI: 0.218-0.744). Furthermore, removing the stent before 14 days may further decrease the risk of UTIs (aOR: 0.311, CI: 0.035- 2.726) (Table 4).
11. The incidence of urological complications post renal transplantation was low and did not reach a statistical difference between the two groups as shown in Table 2

## 4. Discussion

Prophylactic ureteric stenting during kidney transplantation is routinely performed at our center. This approach is shown to reduce major urological complications (MUCs). However, it significantly increases the risk of UTIs post kidney transplantation while the stent is in situ [2, 7, 9, 13–17]. Additionally, stent manipulation at the time of removal by cystoscopy can also introduce more UTIs [18].

In our study, the incidence of UTIs during the first 6 months posttransplantation was 34% which is comparable to the rates reported by other studies [15, 19]. The majority of these UTIs (76.3%) occurred while the stent was still in place and up to two weeks after (i.e., they were “stent-related UTIs”). Beyond that time, UTIs became sporadic and less frequent. This observation suggests that utilizing the concept of “stent-related UTIs” instead of “UTIs in the first 3- or 6-months posttransplantation” maybe more logical and is likely to be a better indicator about the impact of the timing of urinary stent removal on the UTIs. Otherwise, the impact of stent on UTIs can get “diluted with time” and studies may become underpowered to detect significance difference between the two arms [5, 20, 21].

Early removal of ureteric stents has been recommended to decrease UTIs by multiple studies and guidelines [15, 16]. A recent systematic review and meta-analysis including 14 studies including three randomized controlled trials with a total of 3216 kidney transplant recipients, showed significant reduction of UTIs when stents were removed earlier than three weeks (OR: 0.49, 95% CI: 0.33 to 0.75, *p*=0.0009) and without increasing the incidence of urinary leakage compared to delayed removal after 3 weeks [9]. In this study, ESR (<3 weeks) significantly reduced the incidence of UTI related to stent (HR = 0.505, 95% CI: 0.302 to 0.844; *p*=0.009). This beneficial effect remained numerically significant at 6 months (HR: 0.787, CI: 0.474-1.305).

Multiple studies have examined the impact of stent removal at different intervals from renal transplantation including at 4 weeks [6], 3 weeks [5], 2 weeks [4], one week [22, 23], or 5 days [24] post renal transplantation. These studies have shown that the early removal of ureteric stents following kidney transplantation may potentially reduce the incidence of UTI without significant increase of major urological complications. In our study, removing the stent before 21 days posttransplantation decreased UTIs related to stent (aOR: 0.403, CI: 0.218-0.744). Removing the stent before 14 days further decrease the risk of UTIs (aOR: 0.311, CI: 0.035-2.726).

Urinary stents are also risk factors for UTIs recurrence [7]. In our study, the recurrence rate before stent removal was 66%, and it decreased to 46% post stent removal (*p*: 0.06). ESR also led to slight numerical decrease in the rate of MDRO (33% versus 47%; *p*: 0.2). This observation requires further study [25].

In our study, UTIs while the stent was still in situ tended to be more complicated (17.9% versus 4.9%; *p*: 0.019), associated with bacteremia (10.7% versus 0%; *p*: 0.019), and more often resulted in hospitalizations (61% versus 34%; *p*: 0.024). ESR seems to limit the window of these more serious UTIs. This important finding of ureteral stents as a contributing factor of blood stream infections due to UTIs is inline with similar findings observed in few other studies [26–28]. In our cohort, the risk of UTIs with bacteremia significantly decreased once the stent was removed (6 cases before stent removal versus zero after stent removal; *p*=0.019).

For the above reasons, the occurrence of the first UTI post renal transplantation is considered sufficient indication—in our center—to have the stent removed expeditiously once the infection is treated [29]. This is to minimize the risk of recurrence and the risk of more complicated UTIs.

ESR did not increase the urological complications in our study. The incidence of major urological complications (MUCs) post renal transplantation in our recipients (80% living kidney donor) remained low (only 2.1%). There was no statically significant difference between the early versus the late groups. Of note, out of the 55 deceased donor renal transplants in our study, there were no urinary leaks in either early or late groups, and there were only two cases of ureteral stenosis in the late group. ESR in this subgroup is also suggested [30, 31].

Studies have shown that, while there is an estimated cost saving for routine prophylactic stent versus no-stenting of about $200 per patient [6], early ureteric stent removal (8 days versus 15 days) can further reduce UTIs and reduce hospitalization with an estimated cost saving of $2390 per patient [20].

Our study has several points of strength and weakness. Despite the retrospective nature of this study and the small number of patients, this study was able to show the impact of timing of stent removal on the incidence of UTI, recurrence, symptomatology, resistance, and hospitalization in our renal transplant recipients. It was also able to show positive impact of expediting the logistics of urinary stent removal in our center.

## 5. Conclusion

Early ureteric stent removal before 21 days post renal transplantation reduced the incidence of UTIs related to stent without increasing the incidence of urological complication. ESR decreased the risk of UTI recurrence, the risk of complicated UTI, bacteremia, or need for hospitalization due to UTIs.

## Figures and Tables

**Table 1:** Renal transplant recipients' characteristics.

	Total 279	Early 114	Late 165	*p*
Stent removal time (mean ± SD)	35.3 ± 28.0	14.1 ± 4.6	49.9 ± 28.1	<0.001

Age (mean ± SD)	43.4 ± 15.8	44.1 ± 16.2	42.9 ± 15.5	0.544

Gender				
Male	165	68	97	0.902
	59.10%	59.60%	58.80%	
Female	114	46	68	
	40.90%	40.40%	41.20%	

Donor type				0.031
Deceased	55	15	40	
	19.70%	13.20%	24.20%	
Living	224	99	125	
	80.30%	86.80%	75.80%	
Transplant abroad	21	11	10	0.356
	7.50%	9.60%	6.10%	
Preemptive transplant	21	9	12	1
	7.6%	8.00%	7.30%	

Diabetes mellitus				
Type I	25	8	17	1
	26.60%	25.80%	27.00%	
Type II	69	23	46	
	73.40%	74.20%	73.00%	

Induction therapy				
Basiliximab	107	68	104	0.617
	38.40%	60.20%	63.00%	
ATG	172	46	61	
	61.60%	40.40%	37.00%	

Serum creatinine in mmol/L (mean ± SD)				
At 1 month	103.7 ± 47.2	99.7 ± 34.5	106.4 ± 54.2	0.244
At 6 months	99.0 ± 34.0	93.5 ± 21.1	102.7 ± 40.2	0.014
Rejection within 6 months	22	6	16	
	7.90%	5.30%	9.70%	0.416
BK	19	9	10	0.632
	6.80%	7.90%	6.10%	

UTI antibiotic prophylaxis				
TMP/SMX double strength + norfloxacin	70	13	57	<0.001
	25.10%	11.40%	34.50%	
TMP/SMX single strength	209	101	108	
	74.90%	88.60%	65.50%	

Pretransplant urological abnormalities				
Urethral stricture	11	5	6	0.763
	3.90%	4.40%	3.60%	
Neurogenic bladder	10	3	7	0.535
	3.60%	2.60%	4.20%	
Vesicoureteral reflux (VUR)	7	1	6	0.246
	2.50%	0.90%	3.60%	
Emergent stent removal due to stent migration	6	5	1	0.048
	2.1%	1.7%	0.04%	

**Table 2:** Summary of the outcomes of early versus late stent removal.

	Total	Early	Late	*p*
	279	114	165	
UTIs related to stent	74	20	54	0.006
	26%	17.5%	32.7%	

UTIs by 6 months	97	36	61	0.373
	34%	29%	36%	

UTI recurrence in the first 6 months	56	19	37	0.288
	57,7%	52%	60%	

MDRO by 6 months	41	12	29	0.205
	42.2%	33.30%	47.50%,	

UTIs before stent (requiring expedited stent removal)	56	11	45	<0.001
	20%	9.6%	27%	

*Urological complications of renal transplantation*				
Urinary leak	3	0	3	0.272
	1.10%	0.0%	1.80%	
Stenosis/ obstruction	3	1	2	
	1%	0.36%	0.64%	

**Table 3:** UTIs before versus after stent removal (recurrence, symptoms, MDRO, and inpatient treatment).

	UTI		*p*
	Before stent removal	After stent removal	
Total UTI	56	41	

Recurrence	37	19	0.063
	66.1%	46.3%	

Asymptomatic	32	26	0.375
	59.3%	70.3%	

Simple	6	8	0.019
	10.7%	19.5%	
Complicated	10	2	
	17.9%	4.9%	
Complicated with bacteremia	6	0	
	10.7%	0.0%	

MDRO	27	14	0.213
	48.2%	34.1%	

Not treated	5	8	0.024
	9.1%	19.5%	
Treated outpatient	16	19	
	29.1%	46.3%	
Treated inpatient	34	14	
	61.8%	34.1%	

**Table 4:** Unadjusted and adjusted odds ratio for age and sex for UTIs related to stent.

Stent removed	No UTIs	UTIs	OR	95% CI		aOR	95% CI	
≤7 days	7	1	0.378	0.047	3.204	0.311	0.035	2.726
	87.5%	12.5%						

≤14 days	50	8	0.376	0.169	0.836	0.357	0.155	0.82
	86.2%	13.8%						

≤21 days	94	20	0.437	0.244	0.783	0.403	0.218	0.744
	82.5%	17.5%						

≤28 days	120	28	0.431	0.250	0.744	0.392	0.219	0.701
	81.1%	18.9%						

aOR: adjusted OR. Removing the stent before 21 days posttransplantation decreased UTIs related to stent (aOR: 0.403, CI: 0.218-0.744). Removing the stent before 14 days may further decrease the risk of UTIs (aOR: 0.311, CI: 0.035- 2.726).

## Data Availability

The data used to support the findings of this study may be released upon application to the institutional review board at King Abdulaziz Medical City (KAMC), who can be contacted at IRB@ngha.med.sa.
